# Technical Aspects of Deploying UAV and Ground Robots for Intelligent Logistics Using YOLO on Embedded Systems

**DOI:** 10.3390/s25082572

**Published:** 2025-04-18

**Authors:** Wissem Dilmi, Sami El Ferik, Fethi Ouerdane, Mustapha K. Khaldi, Abdul-Wahid A. Saif

**Affiliations:** 1 Department of Control and Instrumentation Engineering, King Fahd University of Petroleum & Minerals, Dhahran 31261, Saudi Arabia; g202318390@kfupm.edu.sa (W.D.); g202212520@kfupm.edu.sa (F.O.); g202113190@kfupm.edu.sa (M.K.K.); awsaif@kfupm.edu.sa (A.-W.A.S.); 2 Interdisciplinary Research Centre for Smart Mobility and Logistics (IRC-SML), King Fahd University of Petroleum & Minerals, Dhahran 31261, Saudi Arabia

**Keywords:** smart logistics, graphical user interface, You Only Look Once (YOLO), object detection, model efficiency, accuracy trade-offs

## Abstract

Automation of logistics enhances efficiency, reduces costs, and minimizes human error. Image processing—particularly vision-based AI—enables real-time tracking, object recognition, and intelligent decision-making, thereby improving supply chain resilience. This study addresses the challenge of deploying deep learning-based object detection on resource-constrained embedded platforms, such as NVIDIA Jetson devices on UAVs and ground robots, for real-time logistics applications. Specifically, we provide a comprehensive comparative analysis of YOLOv5 and YOLOv8, evaluating their performance in terms of inference speed, accuracy, and dataset-specific metrics using both the Common Objects in Context (COCO) dataset and a novel, custom logistics dataset tailored for aerial and ground-based logistics scenarios. A key contribution is the development of a user-friendly graphical user interface (GUI) for selective object visualization, enabling dynamic interaction and real-time filtering of detection results—significantly enhancing practical usability. Furthermore, we investigate and compare deployment strategies in both Python 3.9 and C# (ML. NET v3 and .NET Framework 7) environments, highlighting their respective impacts on performance and scalability. This research offers valuable insights and practical guidelines for optimizing real-time object detection deployment on embedded platforms in UAV- and ground robot-based logistics, with a focus on efficient resource utilization and enhanced operational effectiveness.

## 1. Introduction

With advances in computer vision and deep learning technologies, object detection has become a crucial tool for several applications ranging from surveillance and retail analytics to industrial automation and logistics, all based on the use of unmanned aerial vehicles (UAVs). However, the widespread adoption of deep learning-based object detection is often hindered by high computational demands, making real-time performance particularly challenging on resource-constrained embedded platforms such as NVIDIA Jetson devices deployed in UAV systems. A key factor influencing the performance of object detection models is the quality of datasets used for training. High-quality, diverse, and well-balanced datasets enable models to generalize effectively, improving detection accuracy under varying conditions. Traditional object detection approaches, such as the Histogram of Oriented Gradients (HOG) and Support Vector Machines (SVM), rely on hand-crafted features and struggle in complex environments [1]. The advent of deep learning, followed by Convolutional Neural Networks (CNNs) [2], allowed models to learn hierarchical feature representations directly from data [3,4,5], which significantly enhanced robustness. The introduction of the You Only Look Once (YOLO) framework revolutionized real-time object detection by reformulating it as a regression problem, leading to high-speed inference without sacrificing accuracy. In successive iterations, YOLOv4, YOLOv5 and YOLOv8 have incorporated enhancements such as anchor-free detection and transformer-based layers [6], further optimizing efficiency and precision.

Although deep learning-based object detection has yielded notable improvements, deploying these methods on embedded systems for UAV applications remains a challenge. Careful consideration is required due to stringent limitations in power consumption, computational resources, and real-time processing capabilities. To the best of our knowledge, these challenges have not been thoroughly investigated in prior studies.

This research makes several key contributions to the field of UAV-based logistics using real-time object detection. Firstly, it provides a comprehensive comparative analysis of multiple variants of YOLO, evaluating their performance on critical metrics such as accuracy, inference speed, and resource utilization in the context of UAV deployment. Secondly, it introduces a novel logistics-focused dataset, specifically curated by combining various public datasets to address the lack of domain-specific data, and includes aerial, warehouse, and transportation-related imagery. Third, it develops a user-friendly interface that facilitates real-time filtering and visualization of detected objects, enhancing the practical applicability of the system. Finally, this work culminates in the creation of practical guidelines for the selection and deployment of deep learning-based object detection models on embedded UAV platforms, guiding the efficient implementation of these systems in real-world logistics scenarios

The remainder of this paper is organized as follows: Section 2 reviews advances in YOLO-based real-time object detection, tracking, and hardware optimization, focusing on their deployment in resource-constrained environments such as embedded systems and UAVs. Section 3 describes the training and evaluation of YOLO models using the custom logistics dataset, the hardware and software setup for UAV-based object detection, and the development of a user interface for real-time monitoring and interaction in logistics applications. The results and discussion are provided in Section 4, followed by the conclusion in Section 5.

## 2. Related Work

Object detection has seen major advancements, becoming a key part of computer vision with applications in areas like traffic management and autonomous robotics. YOLO (You Only Look Once), introduced by [7], revolutionized real-time detection by offering impressive speed without sacrificing accuracy. Unlike earlier models such as R-CNN [8] and Faster R-CNN [5], which relied on region proposals, YOLO uses a single CNN to process the entire image in one pass, making it perfect for real-time applications. Over the years, YOLO has evolved, with newer versions like YOLOv4 [9] and YOLOv7 [10] introducing optimizations such as bag-of-freebies and bag-of-specials to improve both detection accuracy and inference speed [11]. YOLOv5 [12] and YOLOv8 [13,14] have introduced improvements in detection speed, model optimization, and hardware adaptability making them suitable for UAV-based vision tasks and specifically for applications involving object detection such as [15] for agriculture, [16,17] for vision-assisted landing of UAVs. Furthermore, investigations into on-board versus ground control station (GCS) processing have explored trade-offs among latency, detection accuracy, and network transmission constraints. Onboard processing, in which the model operates directly on the UAV hardware, enables immediate real-time decision-making [18], which is particularly critical in time-sensitive scenarios such as detecting moving targets [19]. Similar challenges in real-time performance and the handling of heterogeneous data streams have been addressed through the Streaming Vehicle Access Self-Adaption Model (SVASM), which provides an adaptive and efficient framework for accessing high-volume spatiotemporal IoT sensor data under dynamic network conditions [20].

Recent advancements in optimizing operational efficiency through AI-driven techniques have led to significant improvements in both accuracy and resource utilization. For instance, reinforcement learning has been successfully applied to optimize detection paths and reduce excessive data sampling across various domains [21]. Similarly, other studies have explored optimization techniques in UAV-assisted systems, enhancing performance by addressing challenges such as dynamic environments and resource constraints, particularly in UAV applications that demand both computational efficiency and real-time decision-making [22,23]. Using YOLO with image transmission to GCS improved real-time performance while maintaining accuracy. Tests in [17] showed successful detection across different locations, demonstrating the feasibility of lightweight edge computing for UAV applications. In maritime rescue operations, modular, plug-and-play optimizations have been applied to enhance real-time performance [24]. Likewise, in edge computing, YOLO has been successfully implemented on NVIDIA Jetson devices, demonstrating efficient object detection on low-power hardware [25,26,27,28]. The development of custom datasets plays a crucial role in advancing UAV-based applications in smart mobility and logistics. While numerous public datasets exist for general computer vision tasks, there remains a significant gap in datasets tailored specifically for logistics-related scene understanding. Existing object detection datasets such as COCO [29] and KITTI [30] are not fully representative of UAV-based logistics and smart mobility scenarios. Recent advancements in optimizing operational efficiency through AI-driven techniques have demonstrated significant improvements in both accuracy and resource utilization. For example, applying reinforcement learning can optimize detection paths and reduce the number of sampling points while maintaining high precision. Such optimization strategies are particularly relevant in fields like object detection for UAVs, where both onboard and remote processing face similar challenges related to resource constraints and real-time decision-making. One area where these optimizations are particularly relevant is in object detection for UAVs, especially in applications involving both onboard and remote processing [31,32,33]. Studies on model performance analysis have led to the development of several tools that use ‘black-box’ analysis techniques to diagnose complex models in depth. An important distinction can be seen in the types of ML tools available, with some focusing on training models, evaluating performance, and generating detailed metrics [34,35,36]. On the other hand, tools designed for simpler tasks, such as object detection, focus on making the results easily interpretable for consumers. These tools are less concerned with deep technical analysis and more focused on displaying model predictions in an intuitive format, such as highlighting detected objects within an image. They allow users to interact with the model’s outputs directly, without needing to understand the underlying metrics or model evaluation details.

**Figure 1:**
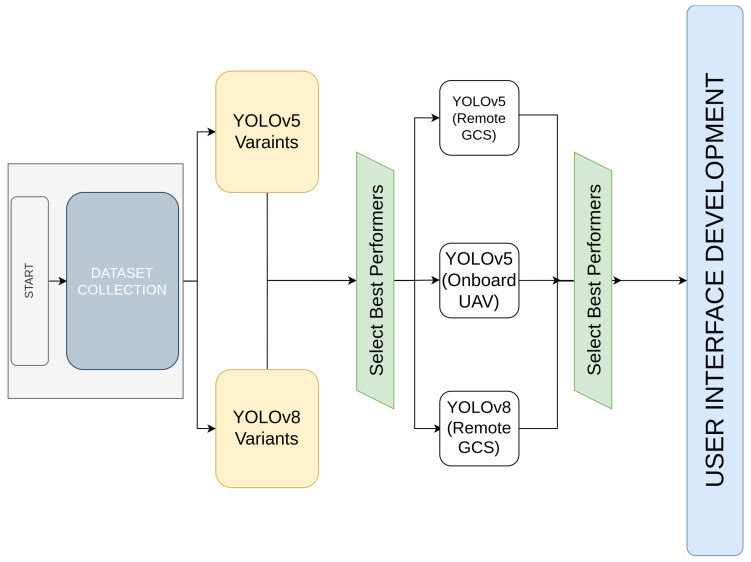
Paper Structure and flow.

This paper explores the integration of UAV object detection with a GUI-based monitoring system to optimize logistics operations. Our study compares YOLOv5 and YOLOv8 to evaluate their effectiveness in detecting objects in real time for UAV applications, with a focus on smart mobility and logistics. The choice between onboard and GCS processing is a fundamental consideration, as the computational resources of UAV hardware have common limitations that can affect both detection speed and deployment scalability. We created a custom dataset by combining multiple online sources to make our testing more realistic. The comparative analysis of YOLO models provided key insights into their respective advantages, which support optimal model selection. A graphical user interface (GUI) for Windows, developed using C#, was designed and implemented to facilitate real-time monitoring of detection results. This has the potential to improve operational efficiency in logistics and mobility applications. A comparative summary highlighting the key contributions of the present study against related research is provided in Table 1, detailing as the YOLO model variant used, the processing type (onboard vs. GCS), logistics focus, and the presence of GUI-based monitoring. An overview of the main steps taken in this paper is shown in Figure 1 to aid understanding of the process followed in this research.

## 3. Materials and Methods

In this section, we present the methodology used to compare onboard and remote inference for UAV-based object detection. The testing environment and dataset are discussed as part of the comparative analysis, followed by details of the experimental setup and the key evaluation metrics: latency, accuracy, and power consumption measurement approach. Finally, the development of the GUI used for real-time monitoring is explained, highlighting its role in the overall framework.

**Figure 2:**
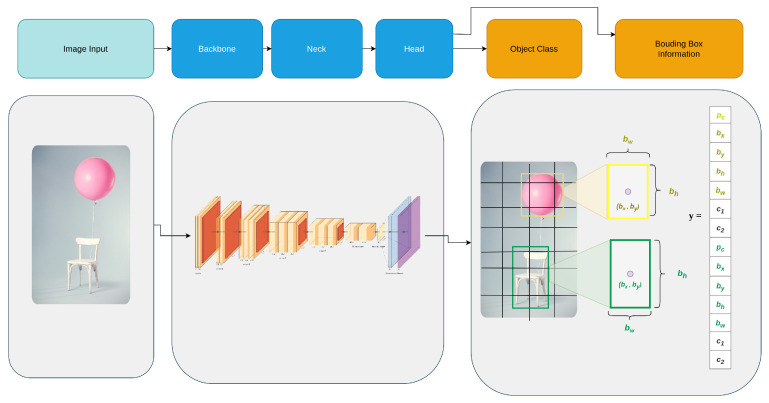
Simplified architecture of the YOLO based detection process, illustrating the object detection pipeline. An input image is processed through the model’s backbone and neck, extracting hierarchical features before passing through the detection head, which outputs bounding boxes around detected objects. The example demonstrates the model’s capability to localize and classify objects within an image.

In this study, we evaluated two different YOLO models, namely YOLOv5 and YOLOv8. Their simplified working pipeline is illustrated in Figure 2. For the object detection task, we selected the COCO128 dataset—a smaller subset of the COCO dataset—as it offers a more efficient alternative. This choice allowed us to speed up the training process while still benefiting from a diverse array of images and annotations. We trained three versions of YOLOv8: large (YOLOv8l), medium (YOLOv8m), and small (YOLOv8s), as well as three corresponding YOLOv5 variants: YOLOv5l (large), YOLOv5m (medium), and YOLOv5s (small). Both YOLOv5 and YOLOv8 are known for their efficiency in real-time object detection, each offering different trade-offs between accuracy and computational requirements.

**Figure 3:**
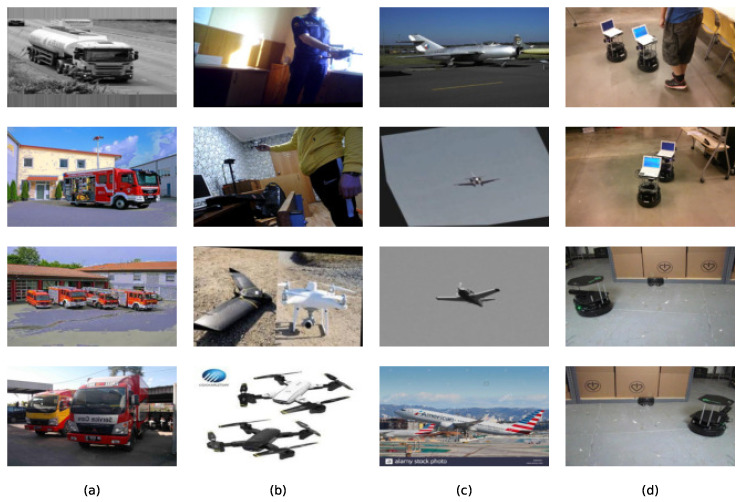
Sample images from the custom Logistics dataset, categorized into four sections. Each column (**a**–**d**) represents a different category: (**a**) samples from a randomly selected logistics-related class [38], (**b**) drone images [41], (**c**) aeroplane images [39], and (**d**) turtlebots [40]. These additional datasets were integrated to enhance diversity and improve model training.

After evaluating the models on the COCO128 dataset, we assessed the accuracy of each model by calculating precision, recall, and mAP50–90, which together provide a comprehensive view of detection performance. These metrics help determine how well each model variant detects objects. We then expanded our focus to logistics applications by developing a comprehensive Custom Logistics Dataset, specifically curated for smart logistics scenarios. This dataset was constructed by merging multiple diverse sources, consisting of approximately 107,034 images from publicly available logistics-focused datasets on Roboflow [38,39,40,41]. After merging these datasets into a unified and cohesive collection—representative samples of which are illustrated in Figure 3—we proceeded to train the top-performing YOLO models, specifically selecting the best-performing YOLOv5 and YOLOv8 versions identified from earlier tests on the COCO128 dataset. The selection criterion emphasized balancing accuracy and computational efficiency, making these models ideal candidates for subsequent evaluation on our logistics-specific dataset. By evaluating model performance on the newly established Custom Logistics Dataset, we aim to determine which model offers the optimal balance between accuracy and computational cost—a critical factor for practical deployment in real-time smart logistics applications.

**Figure 4:**
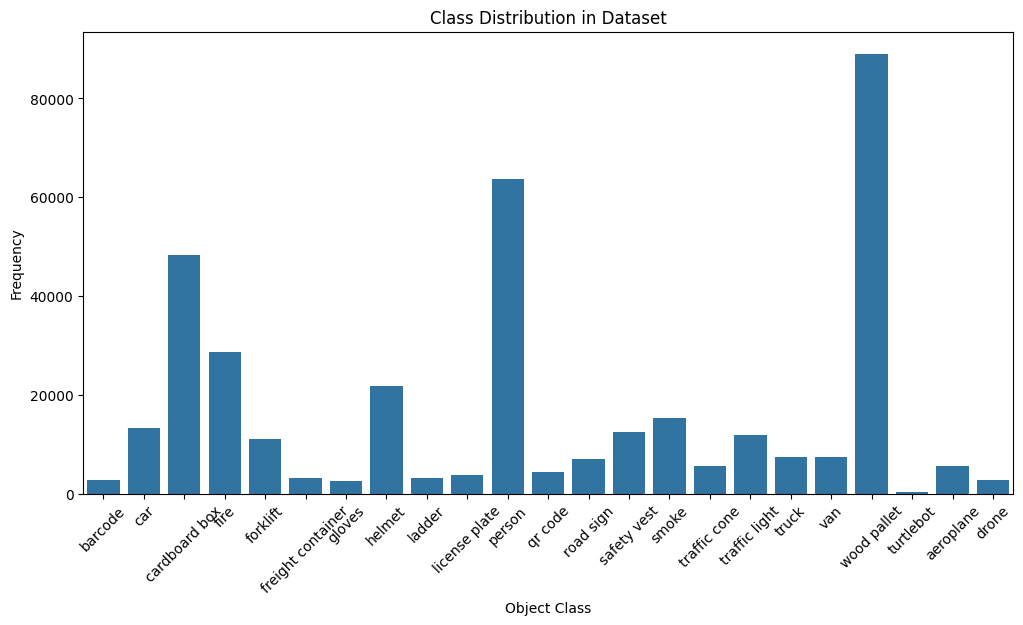
Class distribution for the Custom Logistics Dataset.

The bar plot in Figure 4 illustrates the class distribution. We observe some imbalance, with wood pallets having the highest number of images. Personnel, cardboard boxes, and helmets follow in descending order. This distribution reflects real-world logistics scenarios, where wood pallets are more prevalent than safety gear or emergency supplies.

**Figure 5:**
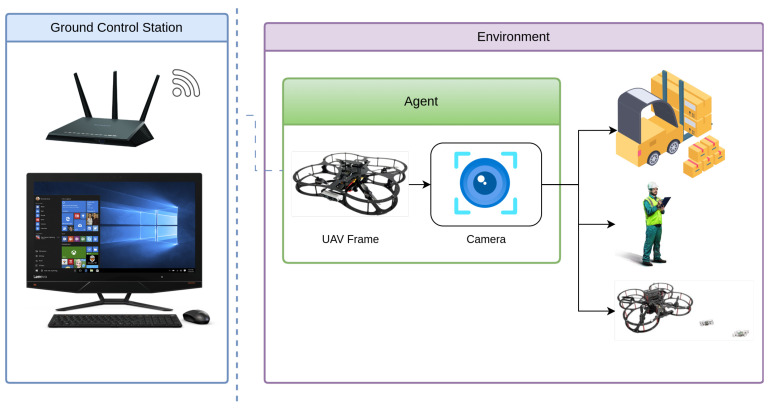
System Overview of the UAV-Based Object Detection Framework in a Logistics related Environment.

**Figure 6:**
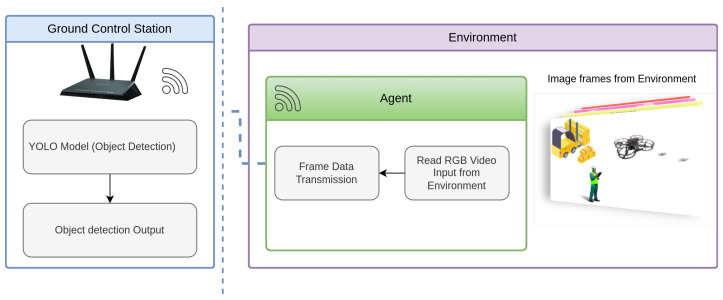
Illustration of the UAV equipped with Intel RealSense RGB camera. The UAV captures visual data from environment, which is transmitted over WIFI to GCS for processing and inference.

Figure 5 illustrates the UAV’s operational setup for object detection in a logistics related indoor environment. The UAV captures real-time video input through its onboard camera, which serves as the primary visual sensor for YOLOv-based object detection. The environment includes key object classes from the logistics dataset—person, drone, airplane. With the help of GCS that facilitates System control for the Qdrone 2 as primary function and real-time monitoring, demonstrating the integration of aerial perception for automated logistics operations, the system is outlined in Figure 6. This visual depiction emphasizes the practical application of both onboard and remote processing systems in a real-world context, helping to contextualize the subsequent performance evaluation. during the tests, the captured images are then processed onboard and on the GCS, enabling a direct comparison of the two methods. For the onboard setup, we measure the inference time and Power consumption. For the remote setup, the latency is measured by accounting for the time taken to transmit the image to the GCS, process it.

### 3.1. Hardware and Software Setup

For this analysis, we used the NVIDIA Xavier NX development kit, which is well-suited for AI tasks, paired with the QDrone2 UAV quadcopter. The Qdrone2 features an Intel RealSense RGB camera, which captures images that are processed by the onboard GPU. the ability to handle the data processing locally, can minimize latency and supports efficient real-time object detection and tracking. In addition, we also evaluated a remote processing configuration using a PC workstation equipped with GPU acceleration, specifically an NVIDIA GeForce RTX 3060, to handle video processing remotely.The PC workstation features a 12th Gen Intel Core i7-12700 processor, 32GB of RAM, and runs Windows 11 Pro with CUDA 12.6 support. This system is used for comparison to assess the performance of the onboard setup against a more powerful remote setup. Building on the analysis of inference time, accuracy, and throughput in both onboard and remote settings, the next step involves examining the software and deployment configurations used for these tests. For the software implementation, we leveraged Python and the Ultralytics YOLO repository, a well-established framework for efficiently loading and running YOLO models.

### 3.2. Model Training and Integration

The combined logistics dataset includes 107,034 labeled images, it was then split into three sections: 9854 images (10%) for testing, 19,704 images (20%) for validation, and 72,962 images (70%) for training. This split supports effective model training and provides an unbiased performance evaluation on unseen data. With this structured dataset, we can now proceed to evaluate the impact of model performance, resource utilization, and inference time in real-world deployment scenarios. This will help us identify the optimal model configuration, balancing both accuracy and efficiency, for logistics-related tasks.

To ensure optimal performance and minimize false positives, the model is configured with specific parameters: The model is trained for 100 epochs with an image size of 640 and a batch size of 16 per GPU, utilizing the Adam optimizer with an initial learning rate of 0.01 and a weight decay of 0.0005 for regularization. Additionally, early stopping with a patience of 10 epochs is enabled, validation is performed throughout training, and model checkpoints are saved.

**Figure 7:**
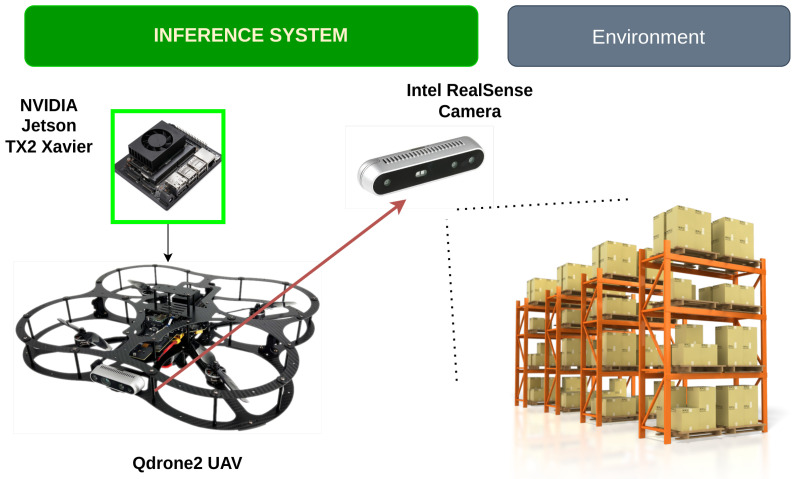
Computer Vision task onboard UAV Jetson TX2.

The YOLOv5s and YOLOv8s models were tested onboard the Qdrone 2 UAV system (Jetson platform) as shown in Figure 7, to evaluate its performance in terms of inference time, accuracy, resource utilization, and power consumption. The model processed video streams at three different resolutions: 320 × 320, 640 × 640, and 1280 × 1280. Each resolution was selected to observe the impact of image size on the processing capabilities of YOLOv5s, specifically focusing on frame rate (FPS) and computational load. The test involved processing frames at each resolution while measuring key metrics such as inference time, FPS, and accuracy. Inference time was recorded to understand the delay in processing each frame, while FPS was tracked to assess the speed of real-time object detection.

**Figure 8:**
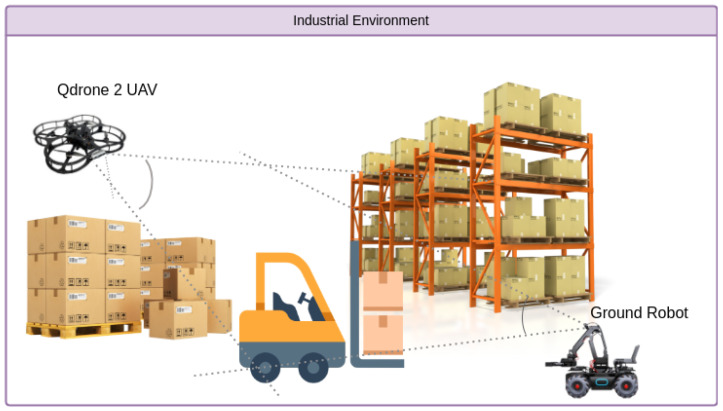
Illustration of the UAV and ground robot perspectives in a warehouse logistics environment. The UAV captures an aerial view, navigating challenges like varying altitudes, speed, and dynamic environments, while the ground robot captures a ground-level perspective, focusing on objects that may be obscured by obstacles.

**Figure 9:**
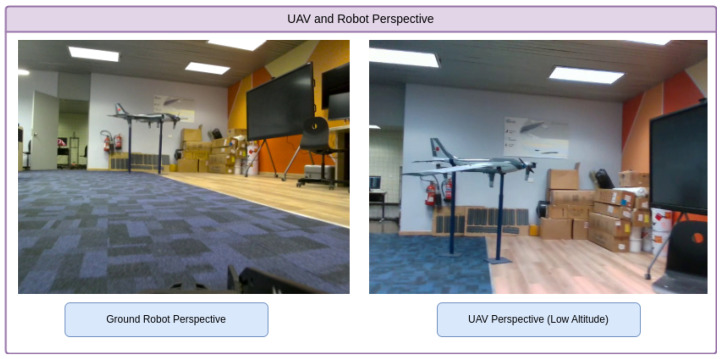
Illustration comparing the UAV’s aerial perspective and the ground robot’s view.

In our testing, we evaluated the model’s performance from two perspectives: the UAV flying through the warehouse and the ground robot navigating the same environment. Figure 8 illustrates data collection from both platforms. The UAV enables real-time object detection and navigation from an aerial perspective. It captures data from a wider angle while flying through the warehouse aisles, handling challenges such as altitude variation, motion blur, and dynamic obstacles. The ground robot, captures a complementary ground-level viewpoint, facing issues like occlusions from shelves and navigating tight spaces. This dual-perspective setup generates a rich and diverse dataset that allows us to thoroughly evaluate the model’s adaptability across real-world logistics scenarios. By capturing both aerial and ground-level views, we can assess how the model performs under varying conditions and sensor configurations, providing valuable insights into the impact of platform type on object detection performance in practical warehouse environments, as shown in Figure 9.

### 3.3. User Interface Development

One important benefit of using a GCS for remote processing, aside from computational performance, is the ability to integrate a GUI for real-time interaction with detection results. In this context, a GUI is developed utilizing C# Windows Forms that improves usability by allowing interactive object selection, manually validate or change detections as necessary, in addition to automated alerts that can be set to warn users when certain detection criteria are met. To ensure real-time processing, video frames are captured using the AForge.Video library [42,43], which ensures low-latency frame collection from the webcam. This reduces delays in visual data processing, which is critical for preserving the accuracy and efficiency of object detection. Within the GUI, identified objects are marked with bounding boxes and confidence values that show the model’s confidence in each detection. Furthermore, the GUI dynamically updates and displays the overall number of chosen objects, offering useful information for applications like inventory management, or crowd monitoring.

**Figure 10:**
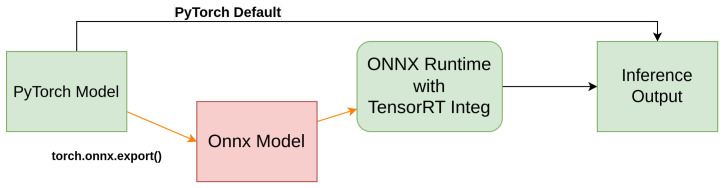
Block diagram illustrating the process of converting a YOLO model from PyTorch (.pt) to the ONNX format and running inference utilizing ONNX Runtime for efficient deployment, and leveraging GPU acceleration with ONNX-TensorRT for optimized performance during inference. This conversion enables seamless integration across different platforms including YoloDotNet for Windows ML model use [44], enhancing the model’s compatibility and resource efficiency.

**Figure 11:**
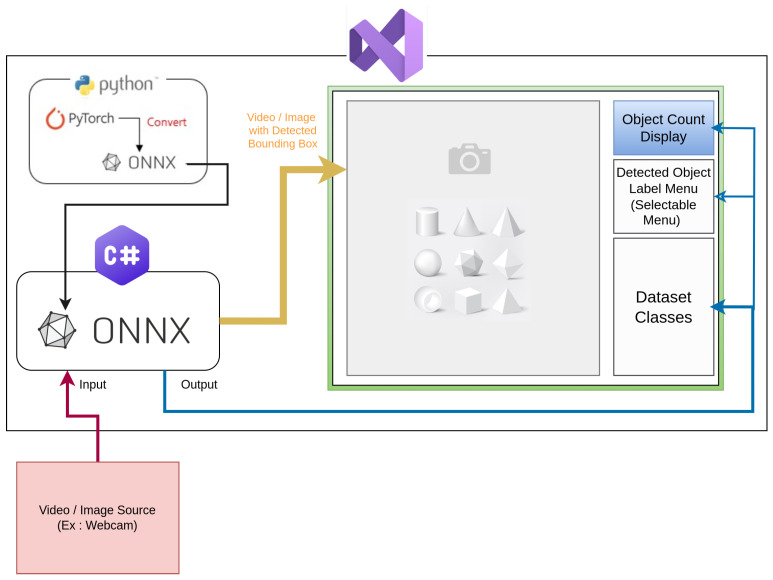
System overview of the dynamic object counting application. The interface displays detected objects and maintains a real-time count, useful for inventory management, crowd monitoring, and wild life Video observation.

As illustrated in Figure 10, the object detection model was initially developed in PyTorch 2.0 and then converted to the ONNX format before implementation. This transformation enabled efficient inference using the ONNX Runtime with TensorRT integration, optimizing the model for real-time processing. Figure 11 depicts the entire system architecture of the designed GUI, including the interaction of various components. The figure also depicts how video frames are processed in real time, beginning with acquisition via camera, progressing via an ONNX-based object identification model, and eventually being shown in the GUI. The detected objects, along with their corresponding labels, are shown within the interface, allowing user interaction for validation and monitoring. The interface also features an object count display, a selectable label menu for detected objects, and dataset class information, further improving user experience and system transparency. The implementation of the video capture and object detection workflow is detailed in Algorithm 1, where the system initializes the model, detects available video devices, and preloads relevant data. The video capture and detection process is managed by the logic outlined in Algorithm 2, which ensures efficient handling of video frames, object detection, and real-time updates of detected objects.
**Algorithm 1** Form Load EventInitialize YOLO model with ONNX fileDetect available video devices**if** No video devices available **then**   Log error   Return**end if**Populate object names from UIPreload item images

**Algorithm 2** Video Capture and Object Detection
**if** videoSource is null **then**   Create new VideoCaptureDevice   Set resolution   Subscribe to NewFrame event
**end if**
**for** each frame captured **do**   **if** frame is being processed **then**     Skip processing   **end if**   Lock object for thread safety   Run YOLO detection   Update detected objects and counts   Unlock object
**end for**



## 4. Results

This section presents the training and deployment results of YOLOv5 and YOLOv8 models. We compare their accuracy, inference speed, and efficiency, selecting YOLOv5s and YOLOv8s for further evaluation. Deployment tests assess onboard and remote processing performance, analyzing power consumption, inference speed, and network impact. Finally, we showcase a user-friendly GUI for real-time object detection monitoring and interaction.

### 4.1. Model Training

Table 2 presents the training results for YOLOv5 and YOLOv8, including their variants (s, m, and l), evaluated on the COCO128 dataset. The comparison includes precision, recall, mean average precision (mAP), and inference time. The YOLOv8l model has the highest accuracy, with an mAP50 of 0.902 and an mAP50-95 of 0.762, but at the expense of longer inference times (165 ms). Although the accuracy difference between YOLOv8s and YOLOv8l is relatively small, the inference speed of YOLOv8s is twice faster (64 ms). For YOLOv5, a similar trend is observed, YOLOv5s has a substantially greater decline in accuracy than YOLOv5l, with MAP50 falling from 0.755 to 0.556. However, YOLOv5s is nearly three times faster (54 ms compare to 147ms for YOLOv8l). Based on these results, YOLOv8s and YOLOv5s have been selected for further training on our custom logistics dataset since they are strong alternatives in cases where speed is a priority and a slight reduction in accuracy is acceptable.

**Figure 12:**
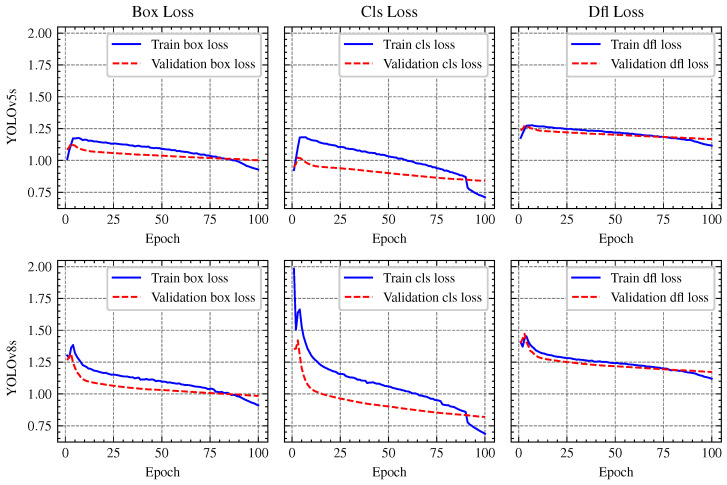
Comparison of YOLOv8 and YOLOv5 Loss Metrics: The **top row** displays the box loss, classification loss, and DFL loss for YOLOv5s, while the **bottom row** shows the corresponding metrics for YOLOv8s. These plots illustrate the differences in convergence rates and overall loss behavior between the two models during training on the custom dataset.

**Figure 13:**
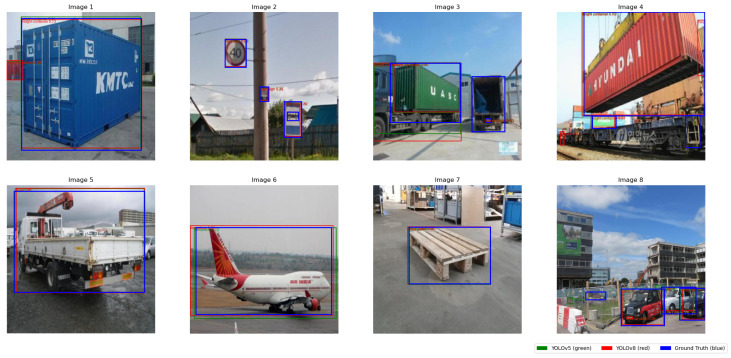
Comparison of object detection results between YOLOv8s and YOLOv5s across a 4 × 2 grid of sample images. Bounding box colors indicate the detection source: green for YOLOv5s predictions, red for YOLOv8s predictions, and blue for ground truth annotations. The images illustrate each model’s detection performance across key object categories, including wooden pallets, personnel, cargo planes, shipping containers, vehicles, and signage.

The training and validation curves for the box loss, classification (cls) loss, and distribution focal (DFL) loss of YOLOv5s and YOLOv8s models are depicted in Figure 12. With loss curves decreasing more rapidly, YOLOv8 demonstrated a higher rate of convergence, suggesting more effective weight optimization. On the other hand, YOLOv5 showed a gradual reduction in loss, requiring more epochs to train. While YOLOv5 maintained stability during training, its improvements in mAP progressed at a slower rate. YOLOv8 demonstrated greater object identification ability by regularly achieving higher mAP values. The performance metrics are summrized in Table 3, indicating that while YOLOv8s achieves a slightly higher accuracy (Box (P) 0.815 vs. 0.814, and mAP50-95: 0.617 vs. 0.608), these gains come at the cost of much greater computational costs. In particular, YOLOv8s shows a greater inference time (56 ms against 23 ms) and requires a longer training period (19.22 h versus 13.67 h for YOLOv5s), suggesting a trade-off between efficiency and accuracy. Figure 13 showcases a 2 × 4 grid of detection results, illustrating that YOLOv8s provides more precise object detections compared to YOLOv5s, particularly in detecting wood pallets, personnel, cargo planes, and cardboard boxes. The details can be see in Table 3.

### 4.2. Model Implementation

In order to assess the models’ deployment and determine their computational demands, their performance is assessed both onboard the UAV and on a GCS. Aspects like Power consumption, CPU and GPU usage, as well as inference time are all evaluated in these tests.

#### 4.2.1. Onboard the UAV

**Figure 14:**
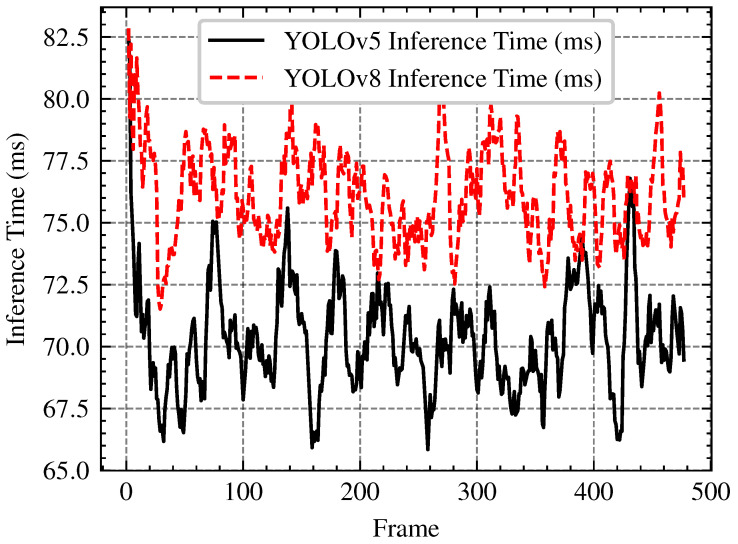
Comparison of Inference Time for YOLOv5s and YOLOv8s Across Frames.

Several factors, including model format (e.g., PyTorch .pt vs. ONNX), input image size, and hardware resource allocation—impact the deployment of YOLO detection models on UAVs using NVIDIA Jetson platforms. These factors can significantly affect computational efficiency, which in turn influences real-time performance on UAVs. The impact of model format has been widely addressed in previous studies [37]. Based on these findings, we selected the ONNX format for its compatibility and efficiency in real-time inference. Furthermore, since input image size is another important factor influencing inference time and overall computational efficiency, we provide a detailed assessment of its impact in the Appendix A. The analysis examines how different image sizes affect YOLOv5’s performance. Additionally, we use the ONNX format with an image size of 640 to evaluate key deployment metrics for YOLOv5s and YOLOv8s, including GPU usage, CPU usage, power consumption (total VDD-GPU-CPU and overall power consumption), and inference time per frame. Figure 14 presents a plot of inference time per frame, while Table 4 summarizes the results for GPU and CPU utilization, as well as power consumption. The results show that YOLOv5 outperforms YOLOv8 in terms of computational efficiency on the NVIDIA Jetson platform, exhibiting lower power consumption, faster inference times, and better GPU utilization. Although YOLOv8 offers improved accuracy, its higher power consumption and fluctuating inference times suggest potential inefficiencies that could impact real-time performance. These findings position YOLOv5 as the preferred choice for real-time applications, particularly on power-constrained edge devices where efficiency and speed are critical for deployment.

#### 4.2.2. Ground Control Station

**Figure 15:**
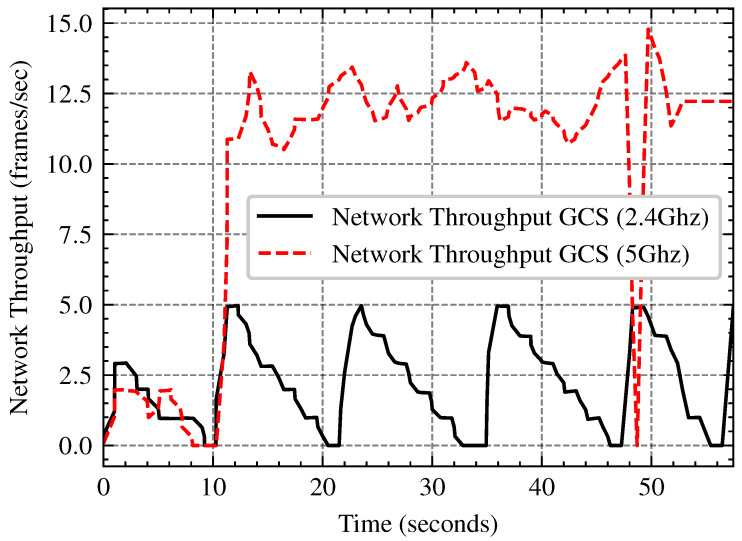
Comparison of Network Throughput Over Time for 2.4 GHz and 5 GHz Frequencies.

**Figure 16:**
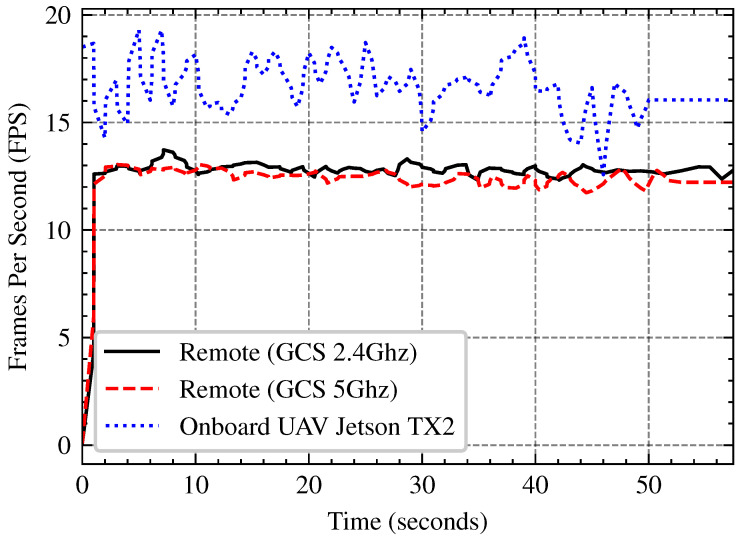
FPS comparison between onboard processing and remote GCS processing over 2.4 GHz and 5 GHz Wi-Fi. Onboard UAV processing (blue dotted line), remote processing over 5 GHz (red dashed line) and 2.4 GHz (black solid line).

**Figure 17:**
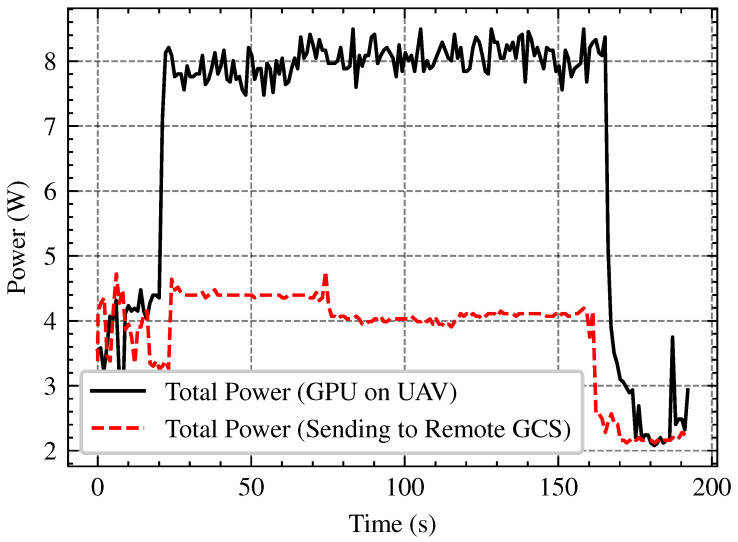
Power consumption comparison between onboard GPU processing (black line) and remote processing via GCS (red dashed line). Onboard processing significantly increases power usage (8 W), while offloading to GCS reduces consumption (4 W), highlighting a trade-off between performance and energy efficiency.

Processing images onboard with an embedded GPU (e.g., NVIDIA Jetson TX2) has power and thermal constraints, nonetheless transferring data to a GCS introduces network latency and bandwidth constraints. In this subsection we compare the performance trade-offs of onboard image processing vs remote processing at the GCS across several network bandwith (2.4 GHz and 5 GHz Wi-Fi). The tests involve evaluating FPS stability, network bandwidth, and power consumption in order to determine the most efficient approach for UAV-based image processing in real-world applications. The network throughput analysis in Figure 15, demonstrates that 5 GHz Wi-Fi outperforms 2.4 GHz by roughly three times, with an average speed of 15 FPS against 5 FPS. As shown in Figure 16 and Figure 17 onboard processing offers the highest FPS (15–18) but at the cost of increased power consumption, while remote processing via GCS over 5 GHz Wi-Fi maintains a relatively stable FPS (12–13 FPS), slightly outperforming 2.4 GHz Wi-Fi. Remote processing conserves power but relies on network stability, making 5 GHz the better option if offloading is necessary.

#### 4.2.3. User Interface

**Figure 18:**
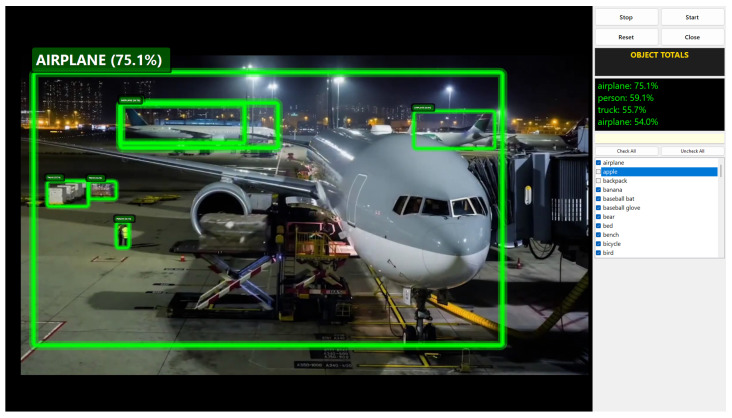
Graphical User Interface (GUI) displaying real-time object detection results. The interface allows users to easily monitor and interact with the model’s performance, providing a clear and intuitive view of detected objects in the environment.

The GUI presented in Figure 18 plays a critical role in improving the system’s usability and performance by enabling users to interact with the model in an intuitive manner. As illustrated in the figure, the GUI provides a user-friendly platform where real-time detection results can be easily accessed, settings adjusted, and the system fine-tuned. One of its most important features is the “Select via Interface” option, which allows users to filter out irrelevant object classes and focus solely on those most relevant to their application. This enhances detection precision while reducing unnecessary computational load, making the system more resource-efficient and practical for real-world use. The feature is particularly valuable in scenarios such as security monitoring or retail analytics, where concentrating on specific objects—such as people or inventory—can significantly improve operational effectiveness.

## 5. Conclusions

This paper presents a real-time object detection system using YOLOv8 within a C# Windows Forms environment. The system is capable of detecting, counting, and tracking objects in video streams, offering a practical solution for applications in retail analysis, security, and industrial settings. In particular, the study explored the real-time performance of the YOLOv5 and YOLOv8 object detection models in the context of smart logistics, focusing on UAV and ground robot platforms. The main goal was to balance the optimization of inference speed and accuracy in these resource-constrained environments. The results showed that onboard processing using Jetson devices has distinct advantages over remote PC-based inference, although the performance varied depending on the model. YOLOv5 offered better accuracy, while YOLOv8 provided faster inference times, making both models suitable for different aspects of logistics operations. To strike the best balance between speed and accuracy, we recommend a hybrid UAV + GCS approach. In this setup, YOLOv5s on the UAV can perform real-time object detection, while uncertain detections are sent to the GCS for verification using a more powerful model like YOLOv8m. This method reduces bandwidth usage by only transmitting uncertain cases and sconserves the UAV’s processing power. At the same time, it ensures higher accuracy when needed, as the GCS can handle more complex computations. However, this approach depends on stable communication between the UAV and GCS, which may pose a limitation in environments with poor connectivity.

For real-time operations where speed is the priority, deploying YOLOv5s or YOLOv8n on the UAV is the most efficient option, as these models offer fast processing with low power consumption. This setup is ideal for scenarios such as collision avoidance in warehouse yards, where rapid decision-making outweighs the need for perfect accuracy. For high-accuracy tasks—such as detailed inventory classification—YOLOv8m or YOLOv8l on the GCS is recommended, particularly when processing complex environments or when detecting small and occluded objects is critical.

A key contribution of this study was the creation of a custom logistics dataset, enabling a more realistic and domain-specific evaluation of the models’ effectiveness in real-world scenarios. Additionally, the development of a graphical user interface (GUI) facilitated dynamic and interactive visualization of detection results, making the system more accessible and applicable to practical logistics operations. The study emphasizes the importance of carefully optimizing both hardware and software to achieve real-time performance, providing insights that can inform future deployments of AI-driven object detection systems in logistics. As models and deployment techniques continue to evolve, these solutions have the potential to scale effectively and operate reliably in resource-constrained environments, thereby enhancing the resilience and adaptability of logistics operations.

In conclusion, the findings from this research highlight that the choice of model and processing method depends on the specific logistics task at hand. Future research should explore further optimization of both edge and GCS-based systems, as well as improvements in communication protocols to ensure reliability and scalability in real-world logistics operations.

## Figures and Tables

**Table 1:** Comparison with Related Work.

Ref.	Model	Onboard vs. GCS	Logistics Focus	GUI-Based
[5]	Faster R-CNN	Not Explored	No	No
[9]	YOLOv4	Not Explored	No	No
[10]	YOLOv7	Not Explored	No	No
[25]	YOLOv8	Edge Computing (Jetson)	Maritime Search & Rescue	No
[31]	YOLOv4	GCS Processing	Agriculture	No
[37]	YOLOv5	GCS Only	Agriculture	No
[24]	YOLOv8	Edge Computing	Maritime Search & Rescue	No
[32]	YOLOv5	UAV Edge Processing	No	No
[28]	YOLOv8	Edge Processing	Smart City	No
**This Work**	YOLOv5, YOLOv8	Both Onboard & GCS	Yes (Logistics & Smart Mobility)	Yes (GUI for Real-Time Monitoring)

**Table 2:** Training results of different variants of YOLOv5 and YOLOv8.

Model	Box (P)	Box (R)	mAP50 (B)	mAP50-95 (B)	Inference Speed (ms)
YOLOv5s	0.701	0.500	0.556	0.427	54 ms
YOLOv5m	0.839	0.586	0.687	0.550	98 ms
YOLOv5l	0.780	0.701	0.755	0.618	147 ms
YOLOv8s	0.841	0.730	0.814	0.646	64 ms
YOLOv8m	0.896	0.804	0.887	0.725	110 ms
YOLOv8l	0.916	0.829	0.902	0.762	165 ms

**Table 3:** Training Metrics comparision of YOLOv5s and YOLOv8s.

Model	N of Epoch	Training Time (h)	Box (P)	Box (R)	mAP50-95	Inference Time (ms)
YOLOv5s	100	13.67	0.814	0.760	0.608	23
YOLOv8s	100	19.22	0.815	0.760	0.617	56

**Table 4:** Mean Performance Metrics for YOLOv5 and YOLOv8.

Model	Mean GPU Util. (%)	Mean CPU Util. (%)	Mean Power VDD CPU GPU CV (mW)	Mean Total Power (mW)
YOLOv5s	65.45	89.13	7154.35	11,907.79
YOLOv8s	56.77	87.77	7368.97	12,199.38

**Figure A1:**
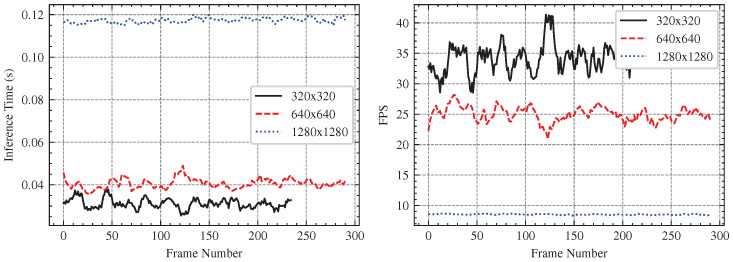
Comparison of inference time (**left**) and FPS (**right**) for different image resolutions. Lower resolutions (320 × 320) achieve faster inference and higher FPS, while higher resolutions (1280 × 1280) result in increased computational load and lower FPS.

## Data Availability

The data presented in this study are available in Roboflow Universe at URL (accessed on 12 April 2025) [https://universe.roboflow.com/large-benchmark-datasets/logistics-sz9jr, https://universe.roboflow.com/cmsc421-final-project/plane-detection-eyzak, https://universe.roboflow.com/kalista/drone-dataset-zxpav-wzg9k-a7ae5, https://universe.roboflow.com/cp214-for-project/turtlebot-detection-2], reference number [38,39,40,41].

## Abbreviations

The following abbreviations are used in this manuscript:

GUI	Graphical User Interface
GCS	Ground Control Station
YOLO	You Only Look Once
UAV	Unmanned Aerial Vehicle
CNN	Convolutional Neural Networks

## Appendix A

Figure A1 clearly show a trade-off between resolution and real-time performance. Lower-resolution images (320 × 320) have the fastest processing speeds, with inference durations ranging from 0.03 to 0.04 s, ensuring minimal delay in real-time applications. Increasing the resolution to 640 × 640 results in a slight increase in inference time to 0.04–0.06 s, while the higherresolution (1280 × 1280) causes a significant increase, around 0.11–0.12 s. This increase in computing effort has a direct impact on FPS, as seen in the right plot, with 320 × 320 resolution achieving the maximum frame rates (30–40 FPS), 640 × 640 performing at a moderate level (20–30 FPS), and 1280 × 1280 struggling to exceed 10 FPS. Higher resolutions may increase detection accuracy, but they also impose computational constraints that can limit real-time responsiveness in UAV-based vision systems.
